# Efficacy of Paromomycin-Chloroquine Combination Therapy in Experimental Cutaneous Leishmaniasis

**DOI:** 10.1128/AAC.00358-17

**Published:** 2017-07-25

**Authors:** Gert-Jan Wijnant, Katrien Van Bocxlaer, Vanessa Yardley, Sudaxshina Murdan, Simon L. Croft

**Affiliations:** a Department of Immunology and Infection, Faculty of Infectious and Tropical Diseases, London School of Hygiene and Tropical Medicine, London, United Kingdom; b Department of Pharmaceutics, UCL School of Pharmacy, London, United Kingdom

**Keywords:** cutaneous leishmaniasis, Leishmania major, Leishmania mexicana, combination therapy, paromomycin, chloroquine

## Abstract

The 4-aminoquinoline chloroquine (CQ) is clinically used in combination with doxycycline to cure chronic Q fever, as it enhances the activity of the antibiotic against the causative bacterium Coxiella burnetii residing within macrophage phagolysosomes. As there is a similar cellular host-pathogen biology for Leishmania parasites, this study aimed to determine whether such an approach could also be the basis for a new, improved treatment for cutaneous leishmaniasis (CL). We have evaluated the *in vitro* and *in vivo* activities of combinations of CQ with the standard drugs paromomycin (PM), miltefosine, and amphotericin B against Leishmania major and Leishmania mexicana. In 72-h intracellular antileishmanial assays, outcomes were variable for different drugs. Significantly, the addition of 10 μM CQ to PM reduced 50% effective concentrations (EC_50_s) by over 5-fold against L. major and against normally insensitive L. mexicana parasites. In murine models of L. major and L. mexicana CL, daily coadministration of 50 mg/kg of body weight PM and 25 mg/kg CQ for 10 days resulted in a significant reduction in lesion size but not in parasite load compared to those for mice given the same doses of PM alone. Overall, our data indicate that PM-CQ combination therapy is unlikely to be a potential candidate for further preclinical development.

## INTRODUCTION

Cutaneous leishmaniasis (CL) is a group of skin infections caused by obligate intracellular protozoa belonging to the genus Leishmania, which are transmitted via the bite of female sandflies. Over 15 different parasite species are responsible for CL in humans, with a diverse clinical spectrum ranging from self-limiting but scarring skin lesions (localized CL) to rarer and more complex forms of CL. These forms can be diffuse (diffuse cutaneous leishmaniasis), chronic (leishmaniasis recidivans), and destructive to the mucosal tissue (mucocutaneous leishmaniasis) (1). The estimated global prevalence is 12 million cases per year in more than 98 countries (the majority of which occurs in Latin America and the Middle East), and more than 350 million people are at risk (2). Despite its increasing incidence (3) and high burden (due to physical disfigurement and related social stigmatization), vaccines and satisfactory treatment options are currently lacking for this poverty-related and neglected tropical disease. A painful and lengthy series of injections of toxic pentavalent antimonials, associated with severe side effects and reduced sensitivity in some species, still remains the first-line therapy after more than 7 decades of clinical use (4). More recent second-line drugs, such the aminoglycoside antibiotic paromomycin (PM), the phospholipid miltefosine (MF), and the polyene antifungal amphotericin B (AmB) (available in deoxycholate salts or lipid nanoparticle formulations), also suffer from similar limitations related to toxicity, efficacy, cost, or an invasive administration route. There is an urgent requirement for new treatments that can eliminate the parasite and safely accelerate lesion healing with minimal scarring and are feasible for use in low-resource health care systems (5, 6). In recent years, combination therapy of commercially available drugs has received more attention as an alternative strategy to develop more effective, lower-dose, and shorter treatments for many infectious diseases, including CL (7–11). With the goal of identifying such an improved therapeutic option for Leishmania major and Leishmania mexicana CL, we investigated the potential of the cheap, safe, and orally bioavailable 4-aminoquinoline chloroquine (CQ) to increase the activities of three standard antileishmanial drugs. The rationale for this approach was based upon evidence that the addition of CQ to treatment regimens for chronic stages of Q fever shortens the duration of therapy and prevents relapses (12, 13). CQ enhances the antimicrobial activity of doxycycline against the causative obligate intracellular bacterium Coxiella burnetii, which resides and multiplies within the phagolysosomes of its macrophage host cells (14). Likewise, CQ improves the effects of specific antibiotics against other intracellular pathogens such as Tropheryma whipplei (Whipple's disease) and persistent Staphylococcus aureus populations in chronic systemic infections (15, 16). Despite the similar macrophage tropism of Leishmania species causing CL, the effect of CQ on the activity of standard antileishmanial drugs has not yet been evaluated. Thus, in this study, our aim was to determine whether combination therapies of PM, MF, and AmB deoxycholate with CQ could be a new approach for the treatment for CL. Promising associations identified during *in vitro* screenings were then assessed *in vivo* by using murine models of L. major and L. mexicana CL. These Old and New World species were compared due to their known differences in drug sensitivities (17) and morphological characteristics of the parasitophorous vacuoles (18).

## RESULTS AND DISCUSSION

We investigated the possible enhancing effect of CQ on the *in vitro* activities of three standard antileishmanial drugs against intracellular L. major and L. mexicana. A specific CQ concentration of 10 μM was selected because of (i) the lack of host cell cytotoxicity (the viabilities of peritoneal exudate macrophages [PEMs] were determined to be 85.8% ± 15.6% and 100% ± 0% in alamarBlue and lactate dehydrogenase [LDH] assays, respectively), (ii) the absence of independent antileishmanial effects (the percent inhibition against L. major and L. mexicana amastigotes was <5% compared to untreated controls), and (iii) the reported enhanced activity of different antibiotics against other intramacrophage pathogens at similar CQ concentrations (14–16). We observed various effects of coincubation with 10 μM CQ for different drugs in standard 72-h drug assays (Table 1). A small but significant (*P* < 0.0001) increase in antileishmanial activity was found for MF against L. major (50% effective concentrations [EC_50_s] decreased from 33.9 ± 5.9 to 10.7 ± 1.8 μM) but not for L. mexicana (from 15.7 ± 1.0 to 10.0 ± 1.6 μM). In the case of AmB, combination with CQ was highly toxic to host cells, and EC_50_s were incalculable. For PM, coincubation with CQ resulted in a significant (*P* < 0.0001) 5-fold decrease in EC_50_s against L. major (from 58.1 ± 6.1 to 11.6 ± 2.4 μM) and reduced EC_50_s against L. mexicana, from >360 μM (i.e., too high to accurately estimate because the concentration was above the maximum drug level tested) to 86.6 ± 17.4 μM (no *P* value was calculable). The improved activity of PM by the addition of CQ was notable because of the known relative insensitivity of L. mexicana to this aminoglycoside antibiotic (19–21). Thus, of the three standard antileishmanial drugs tested, PM in combination with CQ was evaluated further.

**TABLE 1:** *In vitro* activities of miltefosine, amphotericin B, and paromomycin in monotherapy (alone) and in combination therapy with 10 μM CQ (plus CQ) against intracellular L. major and L. mexicana in PEMs after 72 h^*a*^

Organism	EC_50_ (μM)					% infection PEMs (72 h)	
	Miltefosine		Amphotericin B alone	Paromomycin		Untreated	Treated with 10 μM CQ
	Alone	Plus CQ		Alone	Plus CQ		
L. major	33.9 ± 5.9	10.7 ± 1.8*	9.9 × 10^−2^ ± 0.6 × 10^−2^	58.1 ± 6.1	11.6 ± 2.4*	98	97.5
L. mexicana	15.7 ± 1.0	10.0 ± 1.6	9.9 × 10^−2^ ± 0.5 × 10^−2^	>360	86.6 ± 17.4	98.5	96.8

a Data are expressed as means ± 95% CI. *, statistically significant difference in EC_50_s for drugs as monotherapy and CQ combination therapy (*P* < 0.05 by an extra-sum-of-squares *F* test). With amphotericin B plus CQ, there was microscopically visible cytotoxicity toward PEMs. After 72 hours, viability of PEMs treated with 10 µM CQ alone was 85.8% ± 15.6% (alamarBlue assay) and 100% ± 0% (LDH assay).

**FIG 1:**
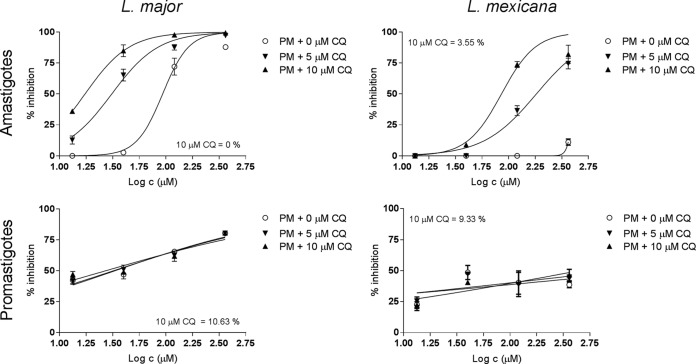
Effect of CQ on the *in vitro* antileishmanial activity of PM against intracellular and extracellular L. major (left) and L. mexicana (right) parasites. For the amastigote drug assays (top row), PEMs were infected with stationary-phase promastigotes and exposed to fixed PM concentrations (13.3, 40, 120, and 360 μM) combined with 0 to 5 to 10 μM CQ for 72 h, followed by microscopic counting of the number of infected macrophages. For the promastigote drug assays (bottom row), exponential-growth-phase parasites were treated identically, and inhibition was evaluated by using the alamarBlue assay. Values are expressed relative to untreated controls (percent inhibition). 10 μM CQ = indicates the percent inhibition at 10 μM CQ.

Next, the concentration dependency of the enhancing effect of CQ on the antileishmanial activity of PM was determined. Fixed concentrations of PM with multiple CQ doses (all ≤10 μM, due to macrophage cytotoxicity at higher concentrations, with the 50% lethal concentration [LC_50_] being 18.1 ± 2.9 μM) were tested against promastigotes and amastigotes of L. major and L. mexicana. Figure 1 shows the corresponding dose-response curves. Increasing CQ concentrations from 0 to 5 to 10 μM did not alter the antileishmanial activity of PM against extracellular promastigotes, with respective EC_50_s of 33.3 ± 8.4, 32.2 ± 8.3, and 27.3 ± 12.3 μM for L. major and >360 μM for all three concentrations for L. mexicana. In contrast, we observed a gradual decrease in EC_50_s against intracellular amastigotes as CQ levels similarly increased (from 91.7 ± 11.2 to 31.0 ± 4.0 to 16.9 ± 1.6 μM against L. major and from >360 μM to 182.1 ± 25.7 to 86.7 ± 22.3 μM against L. mexicana). The mechanism of *in vitro* synergy between CQ and PM to eliminate the intracellular parasites within the parasitophorous vacuoles of the PEMs remains unclear. For the Q fever agent C. burnetii, the mode of action for CQ to enhance the antimicrobial activity of doxycycline, a pH-sensitive antibiotic, is related to its ability to accumulate within the normally acidic macrophage phagolysosomes and alkalinize this pathogen-harboring organelle (14). A similar host-pathogen interaction could be taking place in this setting, as indicated by (i) the presence of lysosomotropic properties of CQ at 10 μM (14–16), (ii) the absence of host cell toxicity and antileishmanial activity of CQ at the chosen concentration, (iii) the specificity of the association synergy for intracellular over extracellular parasites, (iv) the acidophilic nature of Leishmania (22), and (v) the reduced activity of aminoglycoside antibiotics such as PM at acidic pH (23). However, this hypothesis was not confirmed as we did not test whether increased phagolysosomal pH is linked to improved antileishmanial activity.

**FIG 2:**
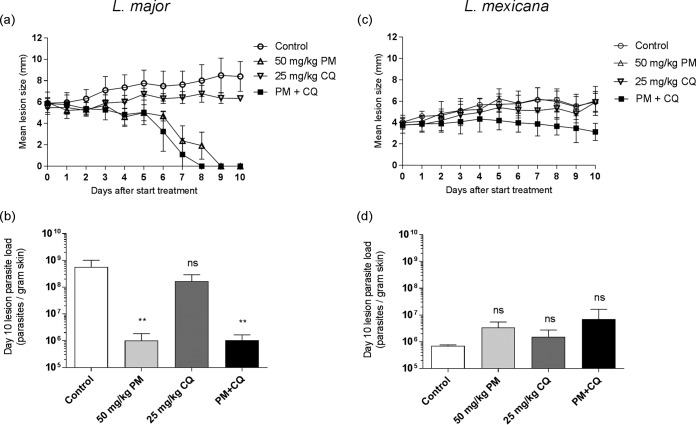
Evaluation of the *in vivo* efficacy of the combination of PM and CQ in murine models of L. major (left) and L. mexicana (right) CL. Female BALB/c mice were infected with stationary-phase promastigotes in the rump above the tail (*n* = 3 to 5 per group). At 12 days (L. major) and 6 weeks (L. mexicana) postinoculation, animals presenting with CL nodules were dosed daily via the i.p. route for 10 consecutive days with PBS (control), 50 mg/kg PM, 25 mg/kg CQ, or a combination of 50 mg/kg PM and 25 mg/kg CQ (PM+CQ). One day after the administration of the last dose (day 10), animals were sacrificed, lesions were harvested, and parasite burden was quantified by DNA-based qPCR. Lesion size evolution (a and c) and lesion parasite load at the end of treatment (b and d) are shown as means and SD. ANOVA (1 way for parasite load and repeated measures for lesion size) followed by Turkey's multiple-comparison tests was used to compare outcomes among the groups. The difference between untreated controls and individual PM-, CQ-, and PM-CQ-treated groups was considered statistically significant if the *P* value was <0.05 (**, *P* < 0.001) or insignificant if not (ns).

Finally, the efficacy of the PM-CQ combination was assessed in murine models of CL caused by the selected Leishmania species. For L. major- and L. mexicana-infected rodents, differences in lesion sizes and day 10 parasite loads among the groups were analyzed (Fig. 2). There was a good correlation between lesion size (Fig. 2a) and parasite load (Fig. 2b) in the L. major BALB/c model. Compared to the untreated controls (phosphate-buffered saline [PBS]), lesion sizes in L. major-infected animals receiving only 25 mg/kg of body weight CQ were slightly smaller (but the difference between the groups was insignificant; *P* = 0.072) but were significantly reduced in the groups receiving 50 mg/kg PM and 50 mg/kg PM plus 25 mg/kg CQ (PM-CQ) (*P* < 0.0001 for both). When mice treated with PM alone were compared to those treated with PM-CQ, lesions in the combination group were smaller and fully resolved (0 ± 0 mm) 1 day earlier (by day 8 versus day 9), but the difference was not statistically significant (*P* = 0.823). The parasite load was not significantly reduced in the CQ group compared to the untreated group (*P* = 0.062) but was significantly lower for PM alone and the PM-CQ groups (*P* = 0.006 for both). No additional reduction in parasite load was found between the PM and PM-CQ groups (1.0 × 10^6^ ± 0.8 × 10^6^ amastigotes per g skin tissue for both), and the difference was insignificant (*P* > 0.999). For the *in vivo* experiment with L. mexicana, lesion sizes (Fig. 2c) again correlated fairly well with parasite loads (Fig. 2d). L. mexicana lesion sizes in rodents treated with PM, CQ, and PM-CQ were not significantly reduced compared to those in the placebo group (*P* > 0.999, *P* = 0.890, and *P* = 0.216, respectively). However, in specific comparisons of the PM and PM-CQ groups at the end of treatment (day 10), the lesion sizes were smaller for the combination group (3.1 ± 0.8 versus 6.0 ± 1.4 mm), and the difference at this time point was significant (*P* = 0.0014). Parasite burdens were similar in the placebo, PM, CQ, and PM-CQ groups (*P* > 0.05 for all differences). No significant difference (*P* = 0.824) between parasite loads in the PM-CQ group (7.0 × 10^6^ ± 9.3 × 10^6^) and the PM-alone group (3.4 × 10^6^ ± 2.2 × 10^6^) was found. Over the course of treatment of L. major- and L. mexicana-infected mice, no events of severe weight loss or adverse drug effects occurred (data not shown).

We conclude that while combination therapy of 50 mg/kg PM combined with 25 mg/kg CQ did not show increased toxicity, there was not significant additional activity compared to those of the component drugs. Against both tested Leishmania species, the association resulted in a small decrease in lesion size toward the end of treatment (which is remarkable for L. mexicana CL due to the known and hereby confirmed unresponsiveness of this species to PM treatment), but the corresponding parasite loads were not significantly reduced. The independent anti-inflammatory properties of CQ rather than a synergistic mechanism with PM might explain this phenomenon, as we also observed a small but not significant suppressive effect on lesion size in controls treated with CQ alone. While the inhibition of proinflammatory cytokine production and release could hinder classic macrophage activation and the consequent elimination of intracellular parasites, this may reduce skin tissue damage and prevent further inflammation-driven lesion proliferation (24–26). Furthermore, antileishmanial activity of CQ in the L. amazonensis CBA mouse ear model has been reported, although this was observed after higher-dose oral treatment (50 mg/kg) over longer periods (5 weeks) (27).

The poor *in vitro-in vivo* translation of the PM-CQ combination might be explained by several factors. First, skin pharmacokinetics could play a role. The daily 50-mg/kg PM regimen over a 10-day period showed efficacy, in agreement with data from previous work with the L. major-BALB/c mouse model (28), indicating that the drug must be bioavailable at the dermal infection site to exert its antileishmanial activity. Based on the extrapolation of data on CQ accumulation in rat skin after intraperitoneal (i.p.) administration (29, 30), the daily 25-mg/kg dose regimen over 10 days should have resulted in the desired dermal micromolar concentrations within the chosen time frame. The ability of CQ to sequestrate in skin has been extensively reported, as it is used in the treatment of cutaneous lupus and is thought to be a factor in adverse reactions such as pruritus and itching (31). Moreover, Leimer and colleagues (16) showed the additional efficacy of flucloxacillin-CQ compared to the antibiotic as monotherapy against S. aureus in a murine systemic infection model after only 2 i.p. doses of 10 mg/kg CQ over a period of 3 days. Hence, while exposure levels of CQ and PM at the target site were likely sufficient to allow theoretical synergy between the drugs, this could not be confirmed experimentally. Second, in the dermis, CQ might not be able to penetrate infected macrophages to interact with PM for the elimination of intracellular parasites. Ionization of CQ in the acidified extracellular fluid present in many tumors is known to limit its passive passage through the membranes of the targeted cancer cells (32). The well-known phenomenon of local acidosis in inflamed tissues (33), such as Leishmania-infected skin (34), could have led to a similar limitation of uptake at this site. Third, there may be *in vivo* antagonistic effects between PM and CQ. This is unlikely because the drugs have not been reported to affect each other's absorption, distribution, metabolism, or excretion (30). Finally, although the *in vitro* intracellular amastigote drug assay using murine macrophages has proven to be a suitable model to predict *in vivo* activity in mice, the lack of biological relevance (macrophage behavior and the presence of many other types of cells and compounds under physiological conditions) might confound this assumption. There is still a need for more complex *in vitro* antileishmanial drug assays to bridge this gap in preclinical CL drug research. Taken together, the variable susceptibilities of L. major and L. mexicana to the tested drugs also highlight the vast challenge in the identification of a single new (combination) treatment active against the plethora of Leishmania species causing CL.

In summary, our data suggest that the high-dose combination of PM and CQ provides only limited enhanced efficacy (mild effect on the evolution of lesion sizes without an additional reduction in parasite burdens) in L. major- and L. mexicana-infected mice compared to PM monotherapy. These findings indicate that further research, such as optimization of the drug dose ratio, into this combination as a novel treatment for CL is not justified.

## MATERIALS AND METHODS

### Drugs.

For the *in vitro* drug assays, stocks of paromomycin sulfate (20 mM [aq]; Sigma, UK), MF (20 mM [aq]; Paladin Inc., UK), amphotericin B deoxycholate (5.2 mM [aq]) (Fungizone; Gibco, UK), and chloroquine diphosphate (10 mM [aq]; Sigma, UK) were prepared, aliquoted, and kept at −20°C until use. From the same original drug batches, solutions in PBS (0.9% NaOH [pH 7.4]; Sigma, UK) were made for the rodent experiments (mean weight per animal of 20 g) at concentrations of 50 mg/kg PM (5.797 mg/ml), 25 mg/kg CQ (4.032 mg/ml), and 50 mg/kg PM plus 25 mg/g kg CQ (coadministration of the same doses).

### Macrophages.

Peritoneal mouse macrophages (PEMs) were obtained from female 8- to 12-week-old CD1 mice. A 2% (wt/vol) starch (VWR, USA) solution in PBS was injected i.p., and PEMs were harvested 24 h later by peritoneal lavage with RPMI medium containing 1% penicillin-streptomycin (PenStrep; Sigma, UK). After centrifugation at 1,500 rpm at 4°C for 15 min, the supernatant was removed, and the pellet was resuspended in minimal essential medium Eagle (MEME; Sigma, UK) with 10% heat-inactivated fetal calf serum (HiFCS; Gibco, UK). The number of cells was estimated by counting with a Neubauer hemocytometer using light microscopy (×40 magnification).

### Parasites.

L. major MHOM/SA85/JISH118 and L. mexicana MNYC/BZ/62/M379 parasites were cultured in Schneider's insect medium (Sigma, UK) supplemented with 10% HiFCS. These parasites were passaged each week at a 1:10 ratio of the existing culture to fresh medium in 25-ml culture flasks without a filter and incubated at 26°C. For infection of macrophages (*in vitro*) and mice (*in vivo*), stationary-phase parasites (as confirmed by light microscopy) were centrifuged for 10 min at 2,100 rpm at 4°C. The supernatant was removed, and the pellet was resuspended in MEME containing 10% HiFCS. The number of cells was estimated by microscopic counting with a Neubauer hemocytometer.

### Cytotoxicity assays.

Macrophages in a 200-μl suspension (4 × 10^5^ macrophages per ml) were allowed to adhere to the bottom of 96-well plates for 48 h and then exposed to specific drug concentrations over 72 h. Cytotoxicity was evaluated by using the alamarBlue assay (Serotec, UK) and a lactate dehydrogenase assay (LDH kit; Promega, UK) to assess metabolism (cell viability) and enzyme leakage through damaged cell membranes (cell death), respectively. After the addition of alamarBlue (10%) to 150 μl of the treated PEM culture, the latter was incubated at 37°C in 5% CO_2_, and viability was measured over a period of 1 to 24 h by fluorescence (SpectraMax M3 plate reader; Molecular Devices) at a wavelength of 530 nm, with a 580-nm emission wavelength and a 550-nm cutoff. Results were expressed as percent viability compared to the untreated controls after correction for the blank signal. In separate wells, an LDH substrate mix was added to 50 μl of the treated PEM culture at a 1:1 ratio. The plates were incubated at room temperature for 30 min on a mechanical shaker with slow rotation. Stop solution (50 μl) was then added, and the absorbance at 490 nm was determined. Results were expressed as percent cell death compared to the positive controls (PEMs treated with 80 μM podophyllotoxin; Sigma, UK) after correction for the blank. An alamarBlue assay (with the same experimental settings as the ones described above for macrophages) was also used to assess the viability of exponential-phase promastigotes during drug assays. Results were expressed as percent inhibition = 100% − *x*% viability (means ± 95% confidence intervals [CI]).

### Seventy-two-hour intracellular antileishmanial drug activity assay.

One hundred microliters of the PEM culture (4 × 10^5^ macrophages per ml) was added to each well of 16-well LabTek culture slides (Thermo Fisher, UK) and incubated for 24 h at 37°C in 5% CO_2_. Host cells were then infected at a 1:3 ratio (L. major or L. mexicana) using 100 μl stationary-phase low-passage-number parasites and further incubated for 24 h at 34°C in 5% CO_2_. On the day of treatment, drug stocks were thawed and diluted to the appropriate concentrations. After confirmation that macrophage infection levels were above 80% (light microscopy), extracellular parasites were removed by washing, and 100 μl of a drug dilution in MEME with 10% HiFCS, alone or supplemented with 10 μM CQ, was added to the infected PEMs. The final drug concentrations were 360 μM for PM, 30 μM for MF, and 0.5 μM for AmB, which were 1:3 serially diluted, resulting in quadruplicates of 4 different concentrations. After incubation for 72 h at 34°C, the medium was removed, and the slides were fixed with 100% methanol for 2 min and stained with 10% Giemsa for 10 min. The number of infected cells was then measured under each treatment condition by microscopically counting 100 macrophages. The percentage of infected cells after treatment was determined and expressed relative to the untreated control (percent inhibition). Dose-response curves and EC_50_s were calculated by using GraphPad Prism version 7.02 software. Results represent means ± 95% CI.

### *In vivo* L. major and L. mexicana models of CL.

Female BALB/c mice around 6 to 8 weeks old were purchased from Charles River Ltd. (Margate, UK). These mice were kept in humidity- and temperature-controlled rooms (55 to 65% and 25 to 26°C, respectively) and fed water and rodent food *ad libitum*. After acclimatization for 1 week, mice were randomized and subcutaneously (s.c.) infected in the shaven rump above the tail with 200 μl of a parasite suspension containing 4 × 10^7^ low-passage-number (*P* < 5), stationary-phase L. major or L. mexicana promastigotes in RPMI medium. Lesion size was measured daily, and treatment was not started until the development of a 3- to 4-mm nodule. Animals were allocated to 4 groups (*n* = 3 for L. mexicana and *n* = 5 for L. major *in vivo* assays) to ensure comparable lesion sizes under each condition. Mice were treated every 24 h with 25 mg/kg CQ, 50 mg/kg PM, or a combination of these drugs at the same doses for 10 days (in PBS, by the i.p. route). The control group received a similar volume (200 μl) of PBS (i.p.). Drug efficacy was evaluated by daily measurements of lesion size and quantification of parasite loads in the infected skin at the end of treatment. Digital calipers were used to determine the mean size of the nodule in 2 dimensions (length and width). Body weight was recorded daily to monitor clinical deterioration due to pathology or systemic drug toxicity.

### Ethics statement.

All animal experiments were conducted under license X20014A54 according to UK Home Office regulations under the Animals (Scientific Procedures) Act 1986 and EC Directive 2010/63/E.

### Parasite DNA extraction from lesions and parasite loads.

The harvested lesion was cut into 1- to 2-mm-long pieces and placed into SureLock microcentrifuge tubes (StarLab, UK) together with 1 ml PBS and 100 mg of 2-mm zirconium oxide beads (NextAdvance, UK). The tissue was ground by using a BulletBlender Storm 24 instrument (NextAdvance, UK) set at maximum speed (setting 12) for 15 min. DNA from a 200-μl volume of the homogenate was extracted by using the Qiagen DNeasy kit for blood and tissue. Twenty microliters of proteinase K and 200 μl tissue lysis buffer were added, mixed, and left to incubate for 1 h in a water bath at 56°C. According to the manufacturer's protocols, DNA was precipitated by using ethanol and transferred to a DNeasy minicolumn, which retained DNA during multiple washing steps until 50 μl was eluted with the appropriate buffer. For the calibration curve standards, this DNA extract from 10^9^ L. major or L. mexicana promastigotes was added to 450 μl water, and consequent serial dilutions ranging from 10^8^ to 10^1^ were made. A previously established quantitative PCR (qPCR) methodology based on the amplification of the 170-bp region in the Leishmania 18S gene (35) was used to quantify the parasite burden in the lesion. Two-microliter DNA extract samples (diluted 1/100) were amplified in 10-μl reaction mixtures in the presence of 5 μl SensiFAST SYBR No-ROX master mix, 0.25 μM probe, and 0.4 μM each primer. Each run included a negative or no-template control where master mix or DNA was replaced by purified water, respectively. Triplicates of standards (10^8^ to 10^1^) and duplicates of unknown samples were included. The tubes were placed into a 72-sample rotor of the RotorGene 3000 instrument, set at 40 cycles at a denaturation setting of 95°C for 5 min followed by a 2-step amplification cycle of 95°C for 10 s and 60°C for 30 s.

### Statistical analysis.

For the outcomes of *in vitro* assays (Fig. 1), the percentage of inhibition was expressed relative to the untreated 72-h control, and the corresponding sigmoidal dose-response curves were established by using a nonlinear fit with variable slope models. Related EC_50_s were compared by using extra-sum-of-squares *F* tests. For *in vivo* experiments (Fig. 2), the differences in lesion sizes and parasite loads among the groups were assessed by using repeated-measures 2-way analysis of variance (ANOVA) and 1-way ANOVA, both of which were followed by Tukey's multiple-comparison test. *In vitro* data are presented as means ± 95% CI, and *in vivo* data are presented as means ± standard deviations (SD). *P* values of <0.05 were considered statistically significant. All analyses were performed by using GraphPad Prism version 7.02.
